# Dr. Borland’s Essay on Milksickness

**Published:** 1846-02

**Authors:** 


DR. BORLAND'S ESSAY ON MILKSICKNESS.

This is a neatly printed pamphlet of 45 pages, octavo, on a subject of much interest. The paper was originally drawn up for the medical society of Memphis, where the author then resided. Dr. Borland does not profess to add anything from original observation to our previous knowledge of the subject, but merely to act the part of a compiler, bringing together and comparing the observations of different individuals. We may remark that his opinion, carefully formed, is, that the disease arises from a *mineral poison*, which may be derived directly from the soil or water, or indirectly through vegetables; that this poison may be imbibed by more than one kind of vegetable, and that in all researches hitherto made, the *cryptogams*—mushrooms—have been too much neglected. The pamphlet displays extensive reading, and is very well written. It is dedicated to Dr. J. R. Buck, of this city.

We cannot but congratulate Dr. Borland on his return to professional engagements after an absence of two years—a return which he thus signalizes. It is always to us a matter of deep regret to see an accomplished physician wandering amid the mazes of politics, or sauntering along its highways and byways—it is a mingling of things divine and human very foreign to our fancy. We trust our friend will for the future let Polk and Clay and the aspirants for the Presidency, together with Texas, Oregon, and California, all go by the board, and turn his attention exclusively to curing disease, and raising soldiers for the State. C.

